# Mechanisms of Action for Small Molecules Revealed by Structural Biology in Drug Discovery

**DOI:** 10.3390/ijms21155262

**Published:** 2020-07-24

**Authors:** Qingxin Li, CongBao Kang

**Affiliations:** 1 Guangdong Provincial Engineering Laboratory of Biomass High Value Utilization, Guangdong Provincial Bioengineering Institute (Guangzhou Sugarcane Industry Research Institute), Guangdong Academy of Sciences, Guangzhou 510316, China; 2 Experimental Drug Development Centre (EDDC), Agency for Science, Technology and Research (A*STAR), 10 Biopolis Road, Chromos, #05-01, Singapore 138670, Singapore

**Keywords:** small-molecule drugs, drug discovery, structural biology, mechanism of action, inhibitors, chemical biology

## Abstract

Small-molecule drugs are organic compounds affecting molecular pathways by targeting important proteins. These compounds have a low molecular weight, making them penetrate cells easily. Small-molecule drugs can be developed from leads derived from rational drug design or isolated from natural resources. A target-based drug discovery project usually includes target identification, target validation, hit identification, hit to lead and lead optimization. Understanding molecular interactions between small molecules and their targets is critical in drug discovery. Although many biophysical and biochemical methods are able to elucidate molecular interactions of small molecules with their targets, structural biology is the most powerful tool to determine the mechanisms of action for both targets and the developed compounds. Herein, we reviewed the application of structural biology to investigate binding modes of orthosteric and allosteric inhibitors. It is exemplified that structural biology provides a clear view of the binding modes of protease inhibitors and phosphatase inhibitors. We also demonstrate that structural biology provides insights into the function of a target and identifies a druggable site for rational drug design.

## 1. Introduction

**Figure 1:**
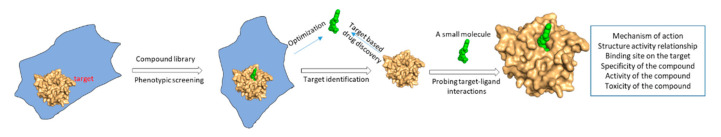
Obtaining molecules to affect the function of a protein. Both phenotypic screening and target-based drug design can be utilized to develop small-molecule compounds.

Drugs are molecules, such as organic compounds and proteins, that regulate a biological process [1,2]. Small-molecule drugs are mainly referring to chemically synthesized compounds with a low molecular weight. A conventional small-molecule drug may have a molecular weight below 500 Da [3], while many developed compounds have a higher molecular weight [4]. Small molecules are able to affect the function of various proteins, including protein–protein interactions, by forming complexes with their targets [5]. Drug discovery of small molecules is a complicated process and requires many techniques and diverse expertise. Small molecules can be obtained through phenotypic screening using cell-based assays in which novel targets can be identified [6,7,8] (Figure 1). These molecules can also be obtained via target-based drug discovery, which usually involves target identification, target validation, assay development, hit identification, hit to lead, lead optimization, candidate selection and later development [9,10]. Medicinal chemistry plays key roles in small-molecule drug discovery as medicinal chemists will make decisions on strategies applied to compound modifications [11,12]. It is well known that there are some obstacles in drug discovery, such as target selection, initial hit identification, lead optimization and efficacies [13,14]. Hit to lead is a critical step in drug discovery and the hits can be achieved from following strategies such as high throughput screening (HTS) [15], fragment identification [16,17], structure-based drug design [18,19,20], artificial intelligence-based drug design [21], known compounds from the published literature [22], the repurposing of approved drugs [10,23] and DNA-encoded library screening [24,25,26]. Biochemical and cell-based assays are able to evaluate the activity of the identified hits while pan-assay interference compounds (PAINS) may give false positive results in the assay [27,28]. Therefore, multiple methods, such as biochemical, biophysical, and structural assays are useful to validate the identified starting compounds [29,30]. Determining modes of action for these starting compounds is critical for lead generation and optimization [31]. Structural biology provides valuable insights to understand the binding modes of the compounds, explore the function of targets and design suitable strategies adopted for compound optimization [32].

## 2. Structural Biology in Drug Discovery

**Figure 2:**
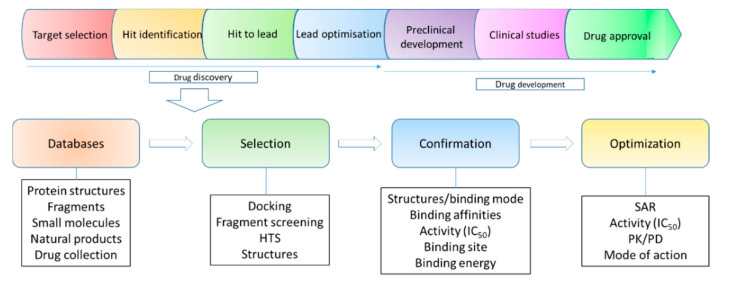
Structural biology in drug discovery. Upper panel shows a simplified flowchart of drug discovery and development. Structural biology plays roles in several steps. The drug discovery stage is summarized as database collection, hit selection and confirmation and the optimization step to get a candidate for preclinical studies. Pharmacokinetic (PK) and pharmacodynamic (PD) are important parameters for a preclinical candidate. Structural biology is critical for generating structures of a target, confirming hits, identifying hits, understanding the ligand binding mode and interpreting SAR.

Structure-based drug design has been widely used in target-based drug discovery, serving as a powerful strategy to design small-molecule drugs [33]. Therefore, structures of targets are always explored at the early stage of a drug discovery project. When a target is identified, the structure of the target can be obtained via different strategies and utilized to predict a ligand binding site, which is critical for understanding molecular interactions and selecting hits using computational methods, such as docking [34]. Structures of target proteins can be obtained through several methods, such as X-ray crystallography [35,36,37], nuclear magnetic resonance (NMR) spectroscopy [38,39], cryogenic electron microscopy (Cryo-EM) [40,41], homology modeling [42] and structure prediction from protein sequences [43]. X-ray crystallography plays critical roles in determining structures of proteins and their complexes at a high resolution [44]. NMR spectroscopy is able to provide both structural, dynamic and ligand-binding information of a target in solution [45,46,47,48]. In recent years, the development of Cryo-EM makes it possible to determine structures of high molecular weight proteins and complexes which is a great contribution in drug discovery [49,50]. As the number of protein structures has increased dramatically, homology modeling can provide reliable structures of many targets (Figure 2).

The early stage of a target-based drug discovery project can be summarized as follows: database preparation, hit selection, hit confirmation and optimization (Figure 2). The database includes compound libraries and structures of a target. The available structure makes it possible to perform druggability analysis and understand the function of the target [51,52]. The structure of a target is also helpful for making suitable strategies in hit selection and lead optimization. For example, a docking or other computational methods can be applied in hit identification, which lowers experimental cost and enhances successful rates in finding hits for further development. Structures of target-hit complexes will favor later compound optimization [53,54]. In fragment-based drug discovery, both X-ray crystallography and NMR spectroscopy can be utilized to perform fragment screening and identify the fragment binding site. Structures of target-fragment complexes are indispensable for deciding a strategy for fragment growth [16,55,56,57]. Structural information of target-ligand complexes enables medicinal chemists to understand the structure–activity relationship (SAR) of the developed small molecules [47,58]. Although a drug can be developed without structural information, the structure of a target is essential for computation-aided drug design [59]. The application of structural biology in drug discovery, especially structure-based drug discovery, has been described in different reviews [32,60,61,62,63]. In this review, we elucidate mechanisms of action for several types of small molecules, such as orthosteric and allosteric inhibitors of viral proteases, revealed by structural studies. In addition, structural information of a protein in complex with its endogenous ligands provides insights into the novel function of the target. The endogenous ligand binding site offers a druggable pocket for developing potent small-molecule inhibitors. The ligands or identified compounds of a target also serve as a chemical probe to explore the function of the target protein in different disease models, which links structural biology with chemical biology and drug discovery.

## 3. New Druggable Sites and Novel Function Derived from Structures

**Figure 3:**
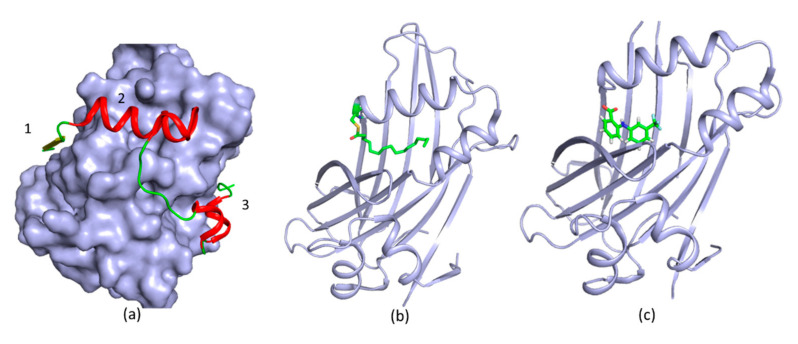
Structures of TEAD in complex with YAP and ligands. (**a**) A structure of TEAD–YAP complex (protein data bank (PDB) access code 3KYS). 1, 2 and 3 indicate the three YAP binding sites on TEAD. TEAD is shown in surface mode and three YAP binding regions are labelled with numbers. YAP is shown in red; (**b**) A structure of palmitoylated TEAD3 (PDB access code 5EMW); (**c**) A structure of TEAD in complex with FA (PDB access code 5DQ8). The ligands are shown in green sticks and bind to TEAD deeply in a pocket.

The Hippo pathway is an evolutionarily conserved signaling pathway affecting apoptosis and cell proliferation [64,65] through the Yes-associated protein (YAP) and its paralog transcriptional coactivator with PDZ-binding motif (TAZ) [66]. YAP/TAZ binds to transcriptional enhancer factor with the TEA/ATTS domain (TEAD) family of transcription factors in the nucleus under certain conditions, which is able to trigger cell proliferation [64,67]. Enhanced YAP/TAZ activities might be relevant to the development of several cancers [68]. TEAD’s interaction with YAP/TAZ is critical for signal transduction. Therefore, the development of inhibitors to disrupt this interaction is an attractive strategy in cancer therapy [69]. Structural studies of the TEAD–YAP complex provide insights into their molecular interactions, while the structures indicate that their binding sites are not favored for developing small-molecule inhibitors, due to the shallow pocket (Figure 3) [70,71,72,73]. Despite this challenge, an effort has been made to develop inhibitors that are able to disrupt TEAD–YAP interactions. Structural studies show that free YAP or TAZ are predominantly unstructured while TEAD is more druggable than YAP as it has three sites binding to YAP (Figure 3) [74]. Some peptides and fragments are able to interact with interface 3 [75,76,77,78,79,80]. Cyclic peptides derived from amino acids of YAP (81–100) were developed to disrupt TEAD–YAP interactions. The optimized peptide exhibited a binding affinity of 15 nM to TEAD1 and a half maximal inhibitory concentration (IC_50_) of 25 nM against protein–protein interactions [76,81]. These types of inhibitors are challenging to be developed into YAP–TEAD disruptors due to their poor drug-like properties. A small molecule-CPD3.1 was identified using molecular docking and it inhibited YAP-induced TEAD1 activity with an IC_50_ of 40 μM. A recent study identified molecules disrupting TEAD–YAP interactions by screening a DNA-encoded library. Two inhibitors against the protein–protein interaction exhibited IC_50_s of 6.75 μM and 5.65 μM, respectively [82]. TEADs contain a conserved hydrophobic pocket in its YAP-binding domain (YBD). High-resolution X-ray structures indicate that this cavity is occupied with a hydrophobic molecule via S-palmitoylation of a cysteine residue (Figure 3) [80,83]. These structures provide insights into a novel function of TEAD [84]. Further study shows that TEAD is able to catalyze self-palmitoylation on a cysteine residue and its palmitoylation is found to be critical for its function [83]. Inhibiting this reaction might affect YAP–TEAD interactions or affect the stability of TEAD. The palmitoylation site of TEAD can serve as a highly druggable site for developing small molecules (Figure 3). Indeed, flufenamic acid (FA) was demonstrated to bind to the pocket of TEAD2 and inhibit TEAD/YAP-dependent transcription in a cell-based assay (Figure 3) [74]. Structural studies on TEADs furnish a clue to explore novel functions of TEADs and identify a druggable site for developing small molecules that inhibit the function of TEADs. It has been noted that challenges still remain to develop a small molecule that is able to compete with the hydrophobic molecule in TEAD.

**Figure 4:**
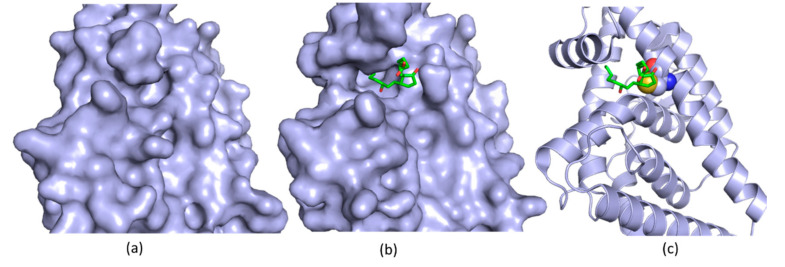
Structures of Nurr1 in the absence and presence of PGA1 (**a**) The structure of free Nurr1 (PDB access code 1OVL). The free protein does not contain a ligand binding pocket; (**b**) The Co-crystal structure of Nurr1 with PGA1 (PDB access code 5Y41). A small molecule binding pocket is present; (**c**) A cysteine residue forms a covalent bond to PGA1 (PDB access code 5Y41). The cysteine residue is shown as spheres and forms a covalent bond with PGA1. All the structures are shown in the same orientation.

A recent study proves that a co-crystal structure of a protein with its ligand is able to expand our knowledge of its function and provides a new strategy to develop inhibitors [85,86]. Nurr1 is an orphan nuclear receptor-a member of NR4A superfamily and important for the development and maintenance of mDA neurons [87]. Accumulated studies suggested that Nurr1 might be a promising target for Parkinson’s disease [88]. However, Nurr1 was considered as an undruggable target as a previous structural study demonstrated that Nurr1 did not have the canonical ligand-binding pocket suitable for binding to a small molecule (Figure 4) [89]. Solution NMR, molecular dynamic and hydrogen/deuterium exchange mass spectrometry studies showed that the putative ligand binding domain in Nurr1 was dynamic and solvent accessible. Conformational changes might be present in the ligand binding site of Nurr1 in the presence of a ligand [90]. Another study on molecular dynamics suggested that Nurr1 contained a putative ligand binding pocket which could be formed through modest structural rearrangements in Nurr1 [91]. Although these studies hypothesized that these family members might have native or synthetic ligands to regulate their function, there is no solid structural information to support this hypothesis. A recent study showed that Nurr1 contained a native ligand which can regulate its function. In this study, prostaglandin E1 (PGE1) was identified to be a ligand of Nurr1 in a cell-based assay using a human neuroblastoma cell line in which tissue extracts exhibited an impact on the transcriptional activation of Nurr1 [85]. The physical interaction between PGE1 and Nurr1 was then confirmed with solution NMR spectroscopy. A titration study demonstrated that PEG1 interacted with Nurr1 in solution. The PEG1 binding site was identified by chemical shift mapping. PEG1 was able to form a covalent bond with Nurr1, as confirmed by mass spectrometry, as an increment of molecular weight was observed when PEG1 was mixed with Nurr1. In a cell-based assay, such interactions were also observed. The structural basis for the molecular interaction between Nurr1 and its ligand was further confirmed by solving the structure of the complex. The co-crystal structure revealed the binding mode of Nurr1 with PGA1—a dehydrated PGE1 metabolite (Figure 4). This structural study revealed that PGA1 formed a covalent bond with a cysteine residue (C566) of Nurr1. Conformational changes were also observed in PGA1 bound Nurr1 and a pocket binding to PGE1 is present in the crystal structure (Figure 4). As predicted by previous NMR and molecular dynamic studies [90], a ligand binding pocket is present in Nurr1. In addition, the endogenous ligand is critical for regulating the function of Nurr1. Further studies indicated that PGE1/PGA1 prominently stimulated Nurr1’s function through direct molecular interactions (Figure 4) [85]. All the above studies on Nurr1 through biochemistry, NMR dynamic analysis, molecular dynamics and co-crystal structures provided solid evidence to demonstrate the presence of the ligand binding pocket in Nurr1. Furthermore, structural studies on Nurr1 paved a new way to understand its regulation by small molecules. More potent small-molecule compounds can be developed by optimizing PGA1 or structure-based drug design upon the achieved structure.

## 4. Different Types of Protease Inhibitors

Viral proteases are attractive targets as they are usually playing important roles in the maturation of viral proteins [92]. We took proteases of flaviviruses, such as Zika virus (ZIKV) and dengue virus, as examples in this review. It is known that ZIKV, West Nile virus and dengue virus are important pathogens threatening human health in tropical and subtropical regions [93,94,95]. The outbreak of ZIKV in recent years affected global population and its infection resulted in serious diseases, such as microcephaly and Guillain–Barré syndrome in adults [96,97,98,99]. ZIKV protease consists of two components, a N-terminal region of NS3 with the catalytic triad and a regulatory region from NS2B critical for NS3 folding and protease activity [100,101,102]. The viral protease is essential for the maturation of nonstructural proteins by cleaving the sites located between several viral proteins. Developing orthosteric inhibitors is a feasible way to combat viruses, while challenges still remain due to the low druggability of the proteases. Structural analysis of proteases in the absence and presence of a ligand indicated that the protease active site is hydrophilic, which is not suitable for developing small-molecule inhibitors [103,104,105,106,107,108,109,110,111,112,113,114]. The following strategies have been applied to overcome this obstacle. Peptidic inhibitors were developed based on substrates of the protease. Allosteric inhibitors and small-molecule inhibitors were also explored. In the development of these inhibitors, structural biology methods, such as X-ray crystallography, NMR spectroscopy and computational modeling, made an impressive contribution to understand the binding modes for the developed inhibitors.

### 4.1. Substrate-Derived Protease Inhibitors

**Figure 5:**
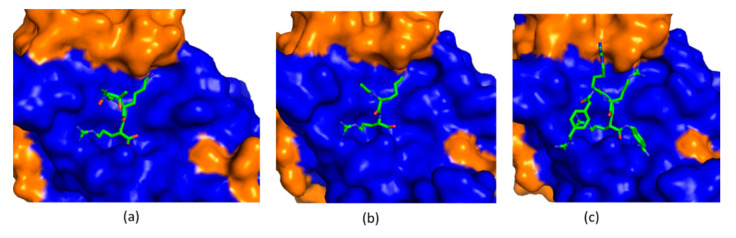
Structures of protease in complexes with its substrate and inhibitors. (**a**) The structure of ZIKV protein in complex with its substrate consisting of Thr–Gly–Lys–Arg (PDB access code 5GJ4); (**b**) A Co-crystal structure of ZIKV protease in complex with a di-peptidic inhibitor (PDB access code 5H6V). This inhibitor exhibited a half maximal inhibitory concentration (IC_50_) of 208 nM [118]; (**c**) A Co-crystal structure of ZIKV protease in complex with a peptidic inhibitor which exhibited a IC_50_ of 1.6 µM (PDB access code 5ZOB). All the structures are shown in the same orientation. The NS2B cofactor region and NS3 protease domain are shown in orange and blue, respectively. The peptide and inhibitors are shown as green sticks.

ZIKV and other flavivirus proteases recognize a peptide sequence containing positive amino acids including Arg or Lys at P1 and P2 positions. Peptidic inhibitors have been developed based on substrates of the protease [115]. The following strategies were adopted to develop potent peptidic inhibitors. First, the length of these peptidic inhibitors was studied. Inhibitors with two or three amino acids exhibited potent inhibitory activity against flaviviral proteases [116]. Second, different warheads were taken to improve the potency. A warhead forming a covalent bond with the protease was shown to be indispensable for this type of inhibitors [109]. Last, peptidomimetics have been explored to develop drug-like molecules for clinical applications. Unfortunately, there are still no peptidic inhibitors entering clinical studies. Structural biology plays important roles in understanding the mechanism of action for these inhibitors [117,118]. In co-crystal structures of flavivirus proteases with a peptide substrate and peptidic inhibitors, the SAR was clearly presented (Figure 5) [104]. For those inhibitors without co-crystal structures, solution NMR spectroscopy and molecular docking approach can provide convincing information to understand their SAR [119].

### 4.2. An Irreversible Small-Molecule Protease Inhibitor

**Figure 6:**
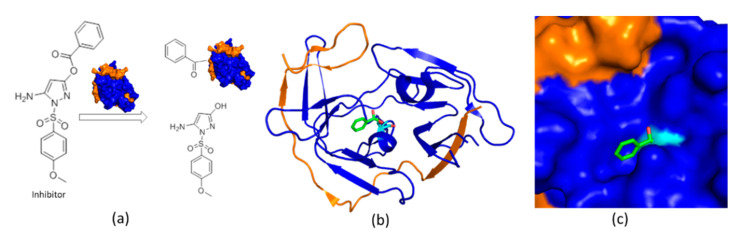
The structure of ZIKV protease in complex with a small-molecule inhibitor. (**a**) The mechanism of action for the small-molecule inhibitor. The chemical structure of the inhibitor is shown. Protease is illustrated in surface mode. NS2B and NS3 are shown in orange and blue, respectively; (**b**) The structure of ZIKV protease-inhibitor complex. The benzoyl moiety is shown in green and S135 is shown in cyan; (**c**) Surface presentation of the complex. The structure was obtained from protein databank with access code (5YOD).

Small-molecule protease inhibitors are of great interest in antiviral development. HTS, structure-based drug design, and *in silico* docking have been utilized to identify potent small-molecule inhibitors [120,121,122,123,124,125], such as HTS identified pyrazole ester derivatives, which are active against proteases of several flavivirus protease [126]. These small molecules are potent protease inhibitors while they are unstable in solution [127] to form a reaction with dengue virus protease [128]. The mechanism of action is not clearly described until a co-crystal structure of ZIKV protein with 5-amino-1-((4-methoxyphenyl) sulfonyl)-1H-pyrazol-3-yl benzoate (compound **1**) (IC_50_ = 1.5 µM) was solved (Figure 6). In the co-crystal structure, only the benzoyl moiety of the inhibitor was observed, forming a covalent bond with S135 of NS3. The hydroxyl–pyrazole moiety of compound 1 was not found to bind to the protease, which was consistent with the results from mass spectrometry. Structural studies also indicated that the benzoyl group stabilized the closed conformation of the protease. The integrity of the compound was demonstrated to be critical for protease binding as fragments derived from the inhibitor did not bind to the protease. Structural analysis of this inhibitor provides solid evidence to understand its mode of action, indicating that it is feasible to develop small-molecule inhibitors against flaviviral proteases [129].

Although the benzoyl group can stabilize the closed conformation of the protease, compound fragments with similar molecular weights of the benzoyl were still not able to inhibit protease enzymatic activity. Fragment-based drug discovery has been applied to develop protease inhibitors, contributing to several fragments identified [130]. Co-crystal structures of fragments with ZIKV protease are solved and these fragments bind to the protease active site [119]. Solution NMR studies showed that these fragments did not suppress conformational exchanges in the protease and they need to be modified for gaining more activities [116,119,131,132]. The identified fragments could serve as a starting point for developing potent protease inhibitors.

### 4.3. Allosteric Protease Inhibitors

**Figure 7:**
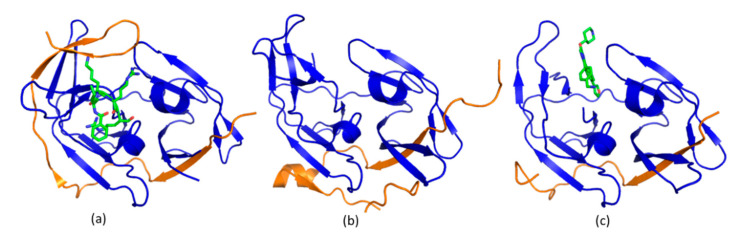
The structure of flavivirus proteases in the absence and presence of inhibitors. (**a**) The structure of protease in the closed conformation (PDB access code 2FP7). The IC_50_ value of the peptidic inhibitor was in micro-molar range [139]; (**b**) The structure of protease in the open conformation (PDB access code 2FOM). The C-terminal region of NS2B cofactor stays away from the active site; (**c**) The structure of protease in complex with an allosteric inhibitor (PDB access code 6MO0). NS2B and NS3 are shown in orange and blue, respectively. The structures of inhibitors are shown in green sticks. All the structures are shown in the same orientation.

Structural studies on flavivirus proteases demonstrated that the protease exists in open and closed conformations. In the open state, the C-terminal region of the NS2B cofactor locates away from the active site to make the enzyme inactive. In the presence of a potent inhibitor or substrate peptides, the C-terminal region of NS2B cofactor forms close contacts with NS3 and substrates/inhibitors, which is referred to as an active/closed conformation. As conformational changes are present in the protease (Figure 7), researchers are interested in developing an inhibitor that is able to stabilize the open conformation which is enzymatically inactive. Unlike those inhibitors targeting the protease active site, allosteric inhibitors were developed by stabilizing the inactive conformation of the protease [133]. With a screening of a library containing compounds targeting lysine specific demethylase 1, an allosteric inhibitor with a IC_50_ of 120 nM was developed (Figure 7) [134]. The inhibitor binding site was confirmed by solving its co-crystal structures. This study is encouraging as the developed inhibitor exhibited anti-ZIKV activity in a cell-based assay [134]. Researchers also pursued other strategies to identify allosteric inhibitors. Based on the crystal structure of dengue virus protease, cysteine mutations were introduced. Using chemical probes specifically reacted with cysteine residues, an allosteric site in the protease was identified [135]. It has been noted that all the structural studies of proteases of ZIKV, dengue virus and West Nile virus took a similar design of protease constructs, which do not contain transmembrane domains of NS2B. The protease might exist only in an active form—the closed conformation under physiological conditions as some factors such as transmembrane regions of NS2B, residues at C-terminus of NS2B and cell membranes could affect conformations of the protease. Therefore, to confirm the binding modes of an allosteric inhibitor of the protease, structural analysis and biophysical methods are utilized to confirm the interaction. In addition, cell-based assays are required to confirm its activity in living cells. Nonetheless, structural biology is critical for understanding the catalytic mechanism for viral protease and provides strategies for developing inhibitors. Quite a few computational-based methods were applied to develop protease inhibitors [136,137,138], which are not described in this review.

## 5. Allosteric Inhibitors for a Phosphatase

**Figure 8:**
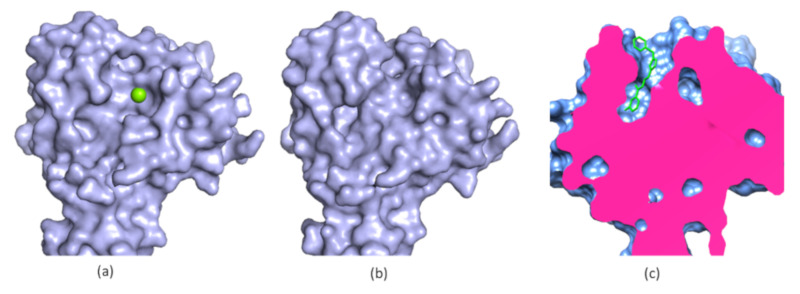
The structure of EYA2 in the absence and presence of an inhibitor. (**a**) The structure of free EYA2 ED (PDB access code 4EGC). Mg^2+^ is present in the structure; (**b**) The structure of inhibitor bound EYA ED (PDB access code 5ZMA). No Mg^2+^ is present in the structure; (**c**) A cut-view of the inhibitor-bound EYA2 ED showing that the inhibitor highlighted in green binds deeply in a pocket.

Structural biology is very important for understanding the function of eye absent (EYA) proteins and provides valuable insights into designing chemical probes to explore their function. EYA proteins (EYA1-4) are a class of proteins binding to the sine oculis homeobox (Six) family of homeobox proteins to activate transcriptions [140,141,142,143]. Six1 and EYA proteins are critical in embryogenesis and they are found to be down regulated in normal adult tissues and overexpressed in some cancers [144,145]. Studies suggested that breaking the interaction of Six1 and EYA proteins is a promising target as this inhibits metastasis in a xenograft model of breast cancer [141]. EYA proteins are multi-domain proteins formed by a transactivation domain (TAD), a domain with phosphothreonine phosphatase (pT-P) activity, a conserved EYA domain (ED) formed by approximately 270 amino acids and a conserved Eya domain 2 (ED2) [146]. The ED domain harbors an intrinsic Tyr phosphatase activity [147,148,149] which might be critical for cancer development [150]. The ED domain is of great interest due to the following reasons. First, it is critical for binding with Six1. As shown in the crystal structure, a helix from Six1 is essential for its molecular interaction with EYA2 ED [141]. The structure of the Six1-EYA2 ED complex shows that it is challenging to develop a small molecule binding to the Six1 binding site, which is a shallow and hydrophilic pocket. An allosteric inhibitor might be able to affect Six1 and EYA interactions. Second, ED of EYA proteins contains a conserved motif from the haloacid dehalogenase (HAD) superfamily via an Asp residue in their active site, while other Tyr phosphatases use a Cys [151]. A structural study showed that the Tyr phosphatase activity of EYA proteins requires Mg^2+^, and this ion is critical for folding of this enzyme [152]. Lastly, the Tyr activity of EYA proteins might be an important target in cancer therapy as three proteins, including H2AX [153,154,155], estrogen receptor β (ER-β) [156] and WDR1 [157] are substrates of EYA proteins. A recent study indicated that inhibiting the Tyr phosphatase activity of EYA2 affected lung cancer migration and invasion, suggesting that suppressing the enzymatic activity might be a strategy in cancer therapy [86]. Structures of phosphatases indicate that the active site is undruggable, resulting from their shallow and hydrophilic surface that favors bindings to negatively charged substrates. Therefore, developing allosteric inhibitors is a feasible strategy to target this type of protein. Structure-based drug design is not straightforward as it is challenging to define a pocket for binding to small molecules based on the available structures of free ED of EYA proteins. To identify potent small-molecule phosphatase inhibitors, a HTS campaign was carried out. A potent EYA2 inhibitor confirmed by biochemical assay was developed with a IC_50_ of 3.0 µM while the inhibitor binding site was not well defined in the absence of a structure [158,159]. A co-crystal structure of EYA2 with the allosteric inhibitor paves a way for developing small molecules against the Tyr phosphatase activity of EYA proteins [86] (Figure 8). The structure revealed the mode of action for the allosteric inhibitor. The inhibitor was shown to bind deeply to a pocket which was not present in a free protein. The inhibitor-binding pocket is formed by some hydrophobic residues and can be used in structure-based design. The co-crystal structure of the complex suggested that inhibitor binding to EYA2 ED caused the movement of a loop region that is critical for Mg^2+^ binding. The inhibitor-bound EYA2 ED does not favor its interaction with Mg^2+^. As Mg^2+¬^ is not present in EYA2, the Tyr phosphatase activity is thus inhibited. The structure also explained the effect of Mg^2+^ on the activity of the inhibitor. Structural studies on EYA2 in the absence and presence of inhibitors provide evidence to understand the function of this enzyme and supported the results from biochemical assays. EYA2 ED might exist in active and inactive conformations. The inactive confirmation does not contain Mg^2+^, which is essential for Tyr phosphatase activity. The active conformation can be induced by the presence of Mg^2+^ and is able to remove the phosphate group from the substrate. The crystal structure also explains the selectivity of the allosteric inhibitor, which is active against EYA2 and not EYA3. As the roles of Tyr phosphatase activity of EYA proteins in cancer/disease development are not well understood, structural studies of ED in the absence and presence of an inhibitor are able to help us understand the enzymatic function of ED. The inhibitor can serve as a specific chemical probe to explore the Tyr phosphatase activity of EYA2 in different disease models. Indeed, the Tyr phosphatase activity of EYA2 was indicated to be critical in the migration of some cancer cell lines. In addition, compound optimization can be carried out on the basis of available structures.

## 6. Characterization of On- and Off-Target Effects

Structures of targets with small molecules are useful for predicting on- and off-target effects of the developed molecules in drug development. On-target effect is defined as the exaggerated responses caused by ligand and target interactions [160]. Although on-target effects can be caused by multiple factors, a covalent inhibitor might have a chance to cause the on-target effect as it forms a tight complex with its target [161]. Off-target effect refers to the adverse effects caused by compound-binding to other targets. Structural bioinformatics enables the prediction of side effects of small molecules [162]. The off-target effect of a small molecule predicts not only the possible side effect but also the phenotypic outcome [163]. With the development of structural biology and computational methods, structures of targets and small molecules can be used to predict the off-target effect, providing a fast and economic way to evaluate the safety of the compounds [164].

## 7. Conclusions

**Figure 9:**
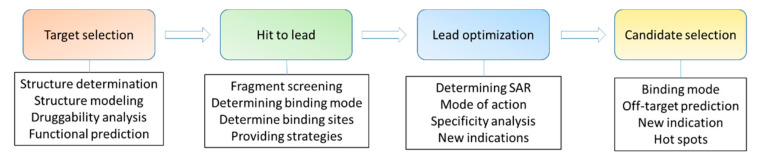
The roles of structural biology in small molecule drug discovery. A simplified flowchart of drug discovery is shown. Structures of a target and its complex will be critical for medicinal chemists to make decisions to modify the compound. A structure of the target-candidate complex might be critical for predicting potential drug resistance. The hot spots identified by structural biology will also be important for developing novel compounds.

Small molecules serve as drugs in therapies and chemical probes to explore the novel function of a target. Structural biology is a critical method to interpret interactions between small molecules and their targets, serving as an important tool in target-based drug discovery (Figure 9). In addition to understanding modes of action of small molecules, structures of targets in complex with their endogenous ligands provide new insights into their function and a way to develop more potent inhibitors. Knowing the structure of a target is critical for its druggability assessment and setting up strategies for hit identification. In the step of hit to lead, structural biology will participate in hit identification, hit confirmation and compound optimization. In lead optimization, structural biology is important for understanding SAR and predicting specificity. In the step of preclinical candidate selection, binding modes of the developed compound determined by structural biology will explain their SAR and might also predict potential drug resistance and off-target effects (Figure 9). Upon an available protein structure, computation-aided compound screening and drug design will also play important roles in drug discovery.

## Abbreviations

PAINS	Pan-assay interference compounds
HTS	High throughput screening
NMR	Nuclear magnetic resonance
Cryo-EM	Cryogenic electron microscopy
SAR	Structure–activity relationship
YAP	Yes-associated protein
PDB	Protein databank
HAD	Haloacid dehalogenase
TEAD	Transcriptional enhancer factor with TEA/ATTS domain
ZIKV	Zika virus
PK	Pharmacokinetic
EYA	Eye absent
PD	pharmacodynamic
Six	Sine oculis homeobox
